# Successful Case of Resection of Cancerous Stomach

**Published:** 1881-08

**Authors:** 


### Successful Case of Resection of Cancerous Stomach.

On April 8th, Dr. Wölfler, assistant to Dr. Billroth, performed resection of a cancerous pylorus on a woman aged 52. The case was regarded as favorable for the proceeding, on account of the mobility of the tumor, which was apparently about the size of a hen's egg. The operation, which lasted two hours, was not followed by fever, nor by vomiting, and the patient was able to take fluid food two days later. On the tenth day, she ate, with good appetite, a veal cutlet, and a fortnight after the operation, was in a most satisfactory condition. The wound in the abdominal wall had healed by first intention, and without any constitutional disturbance.— *Wiener Medizinische Wochenschrift.* Billroth has repeated the operation twice since his first successful case, reported in the *Md. Med. Journ.*, May 1st. Both patients died, the first on the 8th day, the second in about 12 hours.

